# Novel Combination of Silver Nanoparticles and Carbon Nanotubes for Plasmonic Photo Thermal Therapy in Melanoma Cancer Model

**DOI:** 10.15171/apb.2018.006

**Published:** 2018-03-18

**Authors:** Mohammad Ali Behnam, Farzin Emami, Zahra Sobhani, Omid Koohi-Hosseinabadi, Amir Reza Dehghanian, Seyed Mojtaba Zebarjad, Mohammad Hadi Moghim, Ahmad Oryan

**Affiliations:** ^1^Nano Opto-Electronic Research Center, Electrical and Electronics Engineering Department, Shiraz University of Technology, Shiraz, Iran.; ^2^Quality Control Department, Faculty of Pharmacy, Shiraz University of Medical Sciences, Shiraz, Iran.; ^3^Center of Experimental and Comparative Medicine, Shiraz University of Medical Sciences, Shiraz, Iran.; ^4^Pathology Department, Shiraz University of Medical Sciences, Shiraz, Iran.; ^5^Materials Science and Engineering Department, Engineering School, Shiraz University, Shiraz, Iran.; ^6^Pathology Department, School of Veterinary Medicine, Shiraz University, Shiraz, Iran.

**Keywords:** Plasmonic photo thermal therapy, Carbon nanotubes, Silver nanorods, Melanoma cancer, Laser diode

## Abstract

***Purpose:*** Plasmonic photo thermal therapy (PPTT) is a therapeutic method in which the photon energy is rapidly transformed into heat via a series of radiative and non-radiative phenomena to ablate cancer. Plasmonic NPs, such as silver NPs (Ag NPs), have considerable properties in optical absorbance. Furthermore, good thermal conductivity and cell penetration ability of carbon nanotubes (CNTs) could improve the efficacy of Ag NPs for PPTT. Decoration of the multi-walled carbon nanotubes (MWCNTs) with silver has been developed to enhance thermal conductivity of the MWCNT particles.

***Methods:*** The Ag NPs were decorated on the CNTs and the ability of these particles (CNT/Ag NPs) in reduction of melanoma tumor size after PTT was evaluated experimentally. For comparison, the PTT of silver nanorods (Ag NRs) and CNTs were investigated. The melanoma tumor was induced by injection of B16/F10 cell line to the inbred mice. Different NPs were injected into the tumors and then irradiated via laser diode (λ=670 nm, P=500 mW, and I= 3.5 W/cm^2^) at scheduled time.

***Results:*** Monitoring of tumor sizes showed that integration of CNTs with silver could enhance the optical absorption of CNTs and improve tumor destruction in PPTT technique.

***Conclusion:*** The CNT/Ag NPs could act as a potent agent in PPTT method in curing solid tumors.

## Introduction

Cancer is a disease defined by invasive and uncontrolled growth of abnormal cells. Current primary treatments for cancer are surgery, radiation, and chemotherapy. Surgical extirpation is efficacious in primary tumors, but limited to surgical detectable and accessible tumors. Chemotherapy is the other method but limited to the drug dosage forms and their side effects.^1^ Radiation, a complementary method, is used to exterminate the remaining cancer cells after surgery, but it could be harmful to the healthy tissues in the vicinity of the tumor region or in the path of the radiation ray.^1,2^ Recently, genesis of a novel class of photo thermally sensitized agents, nano-scale particles, called plasmonic photo thermal therapy (PPTT), made a revolution in photo thermal therapy (PTT). After entrance of nanoparticles (NPs) into the tumor sites, near infrared (NIR) or visible light excitation of NPs could induce a moderate temperature increment in vicinity of the NPs,^3^ termed as hyperthermia, to destroy the cancer cells clinically.^4,5^ Hyperthermia is generally determined as heating tissues to a temperature in the range of 41-47°C for several minutes.^6^ Tumors are selectively damaged due to their reduced heat tolerance in comparison to normal tissues. One of the specific characteristics of this method is to treat recurrent tumors conveniently while NPs are available in the tumor site. Hyperthermia makes irreversible cell damage by rupturing cell membranes, denaturing proteins, and cell cavitation and degeneration leading to cellular necrosis and apoptosis.^1,7^ Thermal tumor therapies, based on hyperthermia phenomenon,^8^ has been expanded by different kinds of nanomaterials, including gold NPs,^9,10^ silver NPs,^11,12^ and carbon nanotubes (CNTs).^13^

Metal NPs have ample applications in electronics, photonics,^14,15^ chemical sensing, and imaging.^16^ Lately, the plasmonic noble metal NPs have attracted a growing deal of interest to themselves. They have considerable impacts on the visible and invisible spectra, which could improve their application in various approaches such as Raman scattering, radiative rate enhancement,^17^ solar cell,^18^ optical biosensor,^19^ cancer treatment,^20,21^ and biological and medical applications.^22,23^ Plasmonic NPs have been distinguished from other nano-platforms such as semiconductor quantum dots, magnetic NPs,^24^ and polymeric NPs by their distinctive surface plasmon resonance (SPR). This phenomenon is derived from photon confinement to a particle size which improves all the radiative and non-radiative properties of the NPs.^25^ Plasmonic NPs, such as gold and silver, could be adjusted to absorb or scatter light at particular wavelengths in the visible and NIR spectra. Light absorption occurs when the energy of incident photons is dissipated via electron oscillation. Totally, optical absorption and scattering of NPs is related to their size, shape, and material nature.^26-28^ In addition to the unique characteristics of metal NPs, the CNTs have specific physical and chemical characteristics like high aspect ratio, ultra-light weight, high mechanical strength, and high electrical and thermal conductivity^29-31^ which are unique for nanotechnology, electronics, optics and biomedical engineering.^32^ The ability of CNTs in heat energy induction after laser excitation in comparison to other materials is appreciable. CNTs have developed widely in drug delivery systems because they could internalize cells very simply^33^ and operate as vehicles for drugs, antigens, genes, nucleic acids, and imaging agents.^31,34^ Some pharmacokinetic studies have reported that CNTs dispersed by different procedures were free of nonspecific toxic effects in mice.^35,36^ Combination of CNTs and noble metals could open up a new era to enhance the application of CNTs in both drug delivery systems and PTT technique.^37,38^ Therefore, they could be an efficient choice to convert light energy into heat in cancer photothermal therapy.

In the present study, the efficacies of CNTs decorated with Ag NPs (CNT/Ag NP), CNTs and Ag nanorods (Ag NRs) as effective agents for PPTT were evaluated *in vivo*.

## Materials and Methods

### Synthesis procedure

Details of synthesis procedure of the CNT/Ag NPs can be found in our previous article.^39^ Briefly, 100 mg of multi-walled carbon nanotubes (MWCNTs) (Plasmachem, Berlin, Germany) was dispersed in 40 ml deionized water by 30 min ultra-sonication. Number of walls, outer diameter and length of the MWCNTs were 3-15, 5-20 nm, and 1-10 μm, respectively. 40 ml aqueous solution of 0.1 M AgNO_3_ was added drop wise to the prepared suspension. The pH of suspension was adjusted to 6 by adding 0.1 M NaOH solution, and it was again ultra-sonicated for 1 hour. Finally, the resultant CNT/Ag NPs were washed 5 times with deionized water and was centrifuged to be separated from the solution.

The Ag NRs and CNTs were used to be compared with CNT/Ag NPs as agents for PPTT. Ag NRs were a gift from Dr. Sabbaghi’s laboratory, Shiraz University, Shiraz, Iran. To enhance the dispersability of CNT/Ag NPs, Ag NRs, and CNTs in aqueous solution, a layer of polyethylene glycol (PEG) (PEG _1000_, Sigma-Aldrich, St Louis, MO, USA) was coated on them, which enhanced their hydrophilicity and dispersibility.^40,41^ For this, 25 mg of nanoparticles was suspended in 25ml of deionized water containing 250 mg of PEG, separately. The suspensions were ultrasonicated for 3 min and were then stirred at room temperature overnight to allow for wrapping hydrophilic polymer around CNT/Ag NPs, Ag NRs, and CNTs. After stirring, the suspensions were centrifuged at 4000 rpm for 10 min to precipitate the unreacted reagents, and the supernatants were dialyzed and collected for further experiments.

Absorption spectra of light by CNT/Ag NPs, Ag NRs, and CNTs were measured by UV-Vis double beam spectrophotometer (PG instruments Ltd., T80+ UV–Vis spectrophotometer, Lutterworth, UK). The microscopic images were taken by transmission electron microscope (TEM) (Philips Electron Optics, The Netherlands).

### Hyperthermia therapy of tumors

#### Cell culture

The murine melanoma cell line B16/F10 (NCBI C540), from the Cell Bank of Pasteur Institute of Iran, was cultured in RPMI 1640 medium (Shellmax, China) under 5% CO_2_ at 37 °C. The culture medium was supplemented with 10% fetal bovine serum (Shellmax, China), 100 IU/mL of penicillin (Invitrogen, USA) and 100 µg/mL of streptomycin (Invitrogen, USA).

#### Tumor induction and hyperthermia therapy

All applicable international, national, and institutional guidelines for the care and use of animals were followed. This experiment was performed in the Center of Experimental and Comparative Medicine, Shiraz University of Medical Sciences.

25 female C57BL/6J inbred mice (age: 6-8 weeks and weight 25±5 g) were randomly allocated into five equal groups. The animals were housed in ordinary cages and maintained under conventional conditions with 14:10 hour light/dark cycle (lights on at 6:00 A.M), and ambient temperature of 22 ± 2 °C with 50% relative humidity. The animals received a balanced diet. They had access to water ad libitum.

200 µl of the B16/F10 melanoma cell suspension at 2.5×10^6^ cells/ml in culture medium was injected to the mice subcutaneously. This highly metastatic cell line causes melanoma cancer in mice. Sixteen days after inoculation, melanoma tumors grew sufficiently (approximately 1cm^3^) in order to begin treatment. At first, all mice were anesthetized by combination of Ketamine 10% (100-120 mg/kg) and Xylazine 2% (6-10 mg/kg). This injection was performed intramuscularly (IM). After shaving and rinsing, the tumors sizes were measured by a caliper and an ultrasound machine (Ultrasonix SonixOP; Burnaby, BC, Canada). They received treatment as follows:

In Group Ι (CNT/Ag group): CNT/Ag-PEG NPs (1 mg/ml) was injected into the tumor at a dose of 200 µl/cm^3^ (tumor volume).

In Group ΙΙ (Ag NR group): Ag NRs-PEG (1 mg/ml) was injected into the tumor at a dose of 200 µl/cm^3^ (tumor volume).

In Group ΙΙΙ (CNT group): CNT-PEG (1 mg/ml) was injected into the tumor at a dose of 200 µl/cm^3^ (tumor volume).

Group ΙV (Laser therapy group) and group V (Control) did not receive any NPs.

The tumor area in groups Ι, ΙΙ, ΙΙΙ and ΙV were irradiated using a CW laser diode (λ= 670 nm, P=500 mW, and I=3.5 W/cm^2^)^42^ for 15 min daily for four days after the start of the treatment (at first, the mice were anesthetized and then their tumors were irradiated). The Control cases were not irradiated. The tumor sizes were measured daily during the period of the treatment. The tumor volume was calculated according to this formula: [(L/2) * W^2^]^13,43^ (L and W indicate the length and width of tumor). The laser wavelength was chosen according to the optical absorption peak of CNT/Ag NPs in visible-near IR range.

#### Histopathological examination

At the end of experiment, the animals were euthanized by chemical anesthetics overdosing, and the mass were excised for histopathological evaluations. The specimens were processed, and the formalin fixed paraffin embedded (FFPE) blocks were prepared and the slides were stained with Hematoxylin and Eosin (HE). The specimens were evaluated grossly and sampled for microscopic evaluation.

#### Statistical analysis 

The results were presented as mean ± standard deviation (SD). Normality of the distribution was checked by One-Sample-Kolmogorov-Smirnov Test. Differences among the groups were determined using ANOVA and Duncan Post-Hoc Test. All the statistical analyses were done by SPSS^®^ statistical software, version 20.0 (SPSS Inc., Chicago, IL, USA) for Windows^®^. Significance was regarded at *P-values< 0.05*.

## Results and Discussion

### Structural and optical characterization

**Figure 1:**
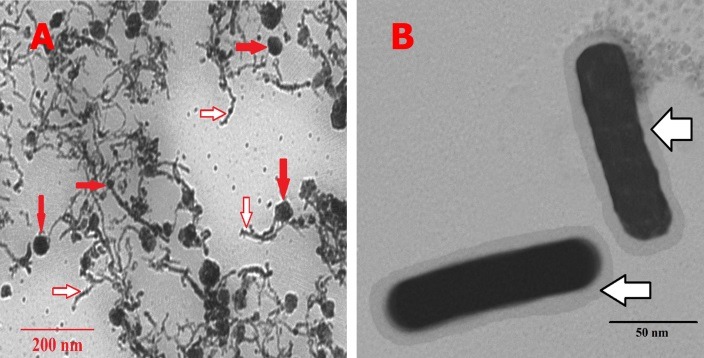
Ultramicrographs of different NPs. (**A**): Ultramicrograph of PEG coated CNT/Ag NPs. (**B**): Ultramicrograph of PEG coated Ag NRs; (open arrows indicate the PEG layer of different NPs and closed arrows indicate the Ag NPs on the surface of the CNTs)

Figure1 shows TEM images of different NPs. According to Figure1 (A), the Ag NPs with the average diameter of 20 nm which were attached to CNTs were synthesized. Also, it was observed that a continuous layer of PEG with the average thickness of a few nanometers was formed on the CNT/Ag NPs. TEM image of Ag NRs coated with PEG is shown in Figure 1(B). This image shows that a continuous layer of PEG with average thickness of 6 nm was formed on Ag NRs.

**Figure 2:**
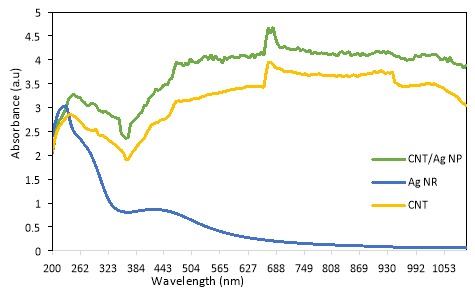
UV-Vis light absorption spectrum of CNT, Ag NR, and CNT/Ag NP

UV-Vis light absorption spectrum of CNT/Ag NPs is given in Figure 2. According to this spectrum curve, the maximum absorption wavelength of CNT/Ag NPs was in the range of 400-1100 nm which is in the visible-near IR range. The broad absorption in the visible-near IR range implies the suitability of the CNT/Ag NPs for PPTT application.

The maximum absorption wavelength of CNT/Ag NPs occurred at 670 nm. These NIR wavelength ranges have considerable optical absorption which could penetrate skin and cells easily^1^ and laser diode wavelength was overlapped with the SPR absorption of CNT/Ag NPs. According to Figure 2, CNT/Ag NPs absorb light in the range of 400-1100 nm significantly better than CNTs, which shows the unique effects of noble metal on enhancing optical absorption of CNTs.

The UV-Vis light absorption spectrum of Ag NRs was measured. Unlike CNT/Ag NPs, Ag NRs showed absorption at UV wavelength range and a weak peak between 360-600 nm. Lack of intense and broad absorption at UV-near IR range, in comparison to that of CNT/Ag NPs, implies unsuitability of Ag NRs for PPTT application. Moreover, UV-Vis light absorption spectrum of CNT is given in Figure 2. Based upon this figure, UV-Vis light absorption of CNT/Ag was improved in comparison to CNT and Ag NR.

### Hyperthermia therapy of tumors

**Figure 3:**
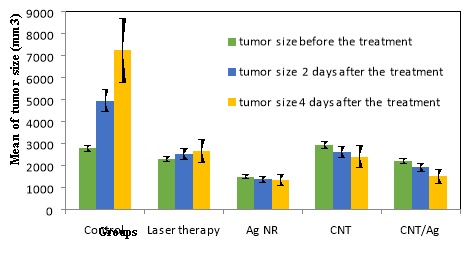
Tumor sizes in different groups at three conditions of measurement (before, two days after, and four days after the treatment with PPTT) (N= 5 in each group and data was shown as mean ± standard deviation).

Tumor sizes were recorded during the experiment. Three prominent situations were reported: before the treatment, two days after the treatment, and four days after the treatment with PPTT technique. As shown in Figure 3, numerical analysis shows that the average tumor size in the Control group increased from 2785.60 mm^3^ before the treatment to7243.93 mm^3^ four days after the treatment, but it decreased significantly in the CNT/Ag group from 2205.12 mm^3^ to1497.52 mm^3^, respectively and in the CNT group from 2920.99 mm^3^to 2403.69 mm^3^, respectively. The sizes of tumors in the Laser therapy group and in the Ag NR group were not significantly different but the growth of tumors was stopped in both groups. The rate of changes in the tumor size was calculated in different groups. The slopes of tumor size against time in the Control, Laser therapy, Ag NR, CNT, and CNT/Ag groups were approximately +1114.59 mm^3^/days, +95.19 mm^3^/days, –40.4 mm^3^/days,–129.32 mm^3^/days, and –176.90 mm^3^/days, respectively (+ indicates increasing and – indicates decreasing of tumor size). The slope of tumor size reduction in the CNT/Ag group was remarkable.

**Figure 4:**
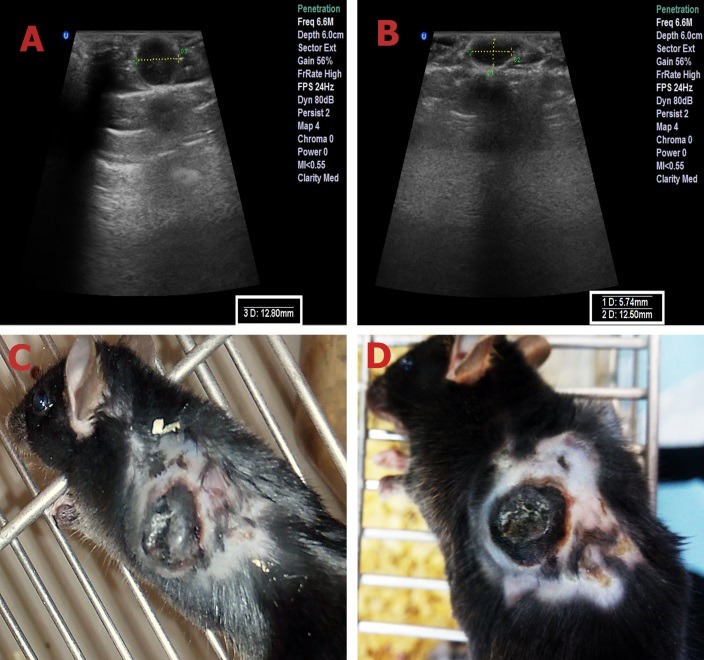
Ultrasonography images and photographs of a cancerous mouse in CNT/Ag group. (**A**, **C**): before the treatment with PPTT. (**B**, **D**): four days after the treatment with PPTT.

**Figure 5:**
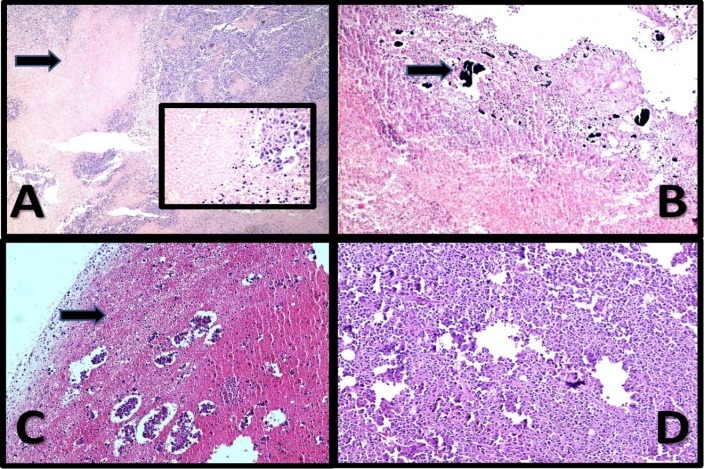
Malignant Melanoma. (**A**): The case treated with laser; Histopathologic evaluation shows the tumor with about 25% necrosis (arrow and inset) (×40-inset ×400) H&E. (**B**): The case treated with Ag NR; Histopathology of the case shows about 45% necrosis in association with NPs (arrow) (×100) H&E. (**C**): The case treated with CNT/Ag NPs; Histopathologic evaluation shows extensive necrosis (about 70% necrosis) with isolated tumor remnant islands (×100) H&E. (**D**): The Control case; Histopathologic evaluation shows sheets of tumor cells with minimal melanin pigments without necrosis (×100)

In addition to these statistical analyses, the ultrasonography images and photographs of a cancerous mouse in the CNT/Ag group showed the healing effect of this technique on shrinking solid tumors, as shown in Figure 4. For more professional deliberation, histopathologic evaluation was done. Gross evaluation of the tumors showed severe necrosis in the CNT/Ag group. Histopathologic evaluation showed nodular subtype in all cases. Necrosis was the most important discriminator between the cases and its percentage was higher in the CNT/Ag group in comparison to the Control group, which is the result of therapy. Mitosis was higher in the Control cases when compared to other cases. There was no evidence of regressive fibrosis, lymphocytic infiltration, vascular invasion or neurotropism in the cases. The histopathologic results are shown in Table 1 and Figure 5.

Plasmonic NPs can absorb NIR light and convert its energy into heat. When NPs absorb light, electrons have to transit from the ground states to the excited states. The absorbed light is transformed into heat via a series of photo physical processes which contain electron-electron relaxation and electron-photon relaxation.^1,2,28^ According to this phenomenon, the heat generated from plasmonic NPs after NIR irradiation can be used for treatment of solid tumors. The tumor cells are more sensitive to elevated temperature than normal cells.^13^ In this study, we used CNTs decorated with Ag NPs (CNT/Ag) to evaluate their ability for tumor size reduction by PPTT technique. The efficacy of these NPs was compared to the effect of Ag NRs and CNTs. In the previous study we observed that irradiation of CNTs in the tumor region caused considerable damage to the melanoma *in vivo.*^13^ Ag NRs have no significant absorption in the NIR range but the absorption of CNT/Ag NPs is very considerable even in comparison with CNTs.

**Table 1:** Histopathologic results of tumor tissues after PTT in different groups of mice

Groups	Necrosis (%)	Mitotic rate
CNT/Ag	~ 70%	<1/mm^2^
CNT	~ 60%	<1/mm^2^
Ag NR	~ 45%	<1/mm^2^
Laser therapy	~ 25%	<1/mm^2^
Control	< 5%	>1/mm2

At first, to improve the dispersibility of NPs in water, the hydrophilic polymer PEG was used to wrap the surface of NPs. Melanoma was induced in mice by injection of B16/F10 cell line. CNT/Ag NPs, Ag NRs, and CNTs were injected into the tumor. The tumor area was irradiated by a 670 nm CW laser diode after injection of NPs. Analyzing the results of this study showed that, in a definite period of irradiation, the CNT/Ag NPs resulted in tumor necrosis more effectively than the CNTs and Ag NRs in the melanoma cancer model. Based upon the attached UV-Vis light absorption spectra, this phenomenon was due to the enhanced optical absorbance of CNT/Ag NPs in comparison to CNTs and Ag NRs in the visible-near IR range. Laser excitation could increase hot electrons temperature rapidly in NPs and consequently resulted in elevated lattice temperature. Therefore, CNT/Ag NPs generated heat more efficiently than CNTs and Ag NRs. Monitoring the tumor size showed that in the groups receiving CNT/Ag NPs, Ag NRs, and CNTs, not only the tumor growth was stopped after irradiation but the tumor size also shrunk. However, in the group that underwent only laser therapy without any pretreatment, just the rate of tumor growth was slower than the Control group. Besides, histopathological examination showed 70% necrosis in the CNT/Ag group which demonstrated high efficiency of the CNT/Ag NPs as agents in PPTT method.

The results confirmed that combination of CNTs and silver could enhance the optical absorption of CNTs and, consequently, improve the tumor destruction in PPTT technique.

## Conclusion

Integration of CNTs with silver increased the optical absorption of CNTs significantly. CNT/Ag NPs induced heat generation and resulted in considerable damage to melanoma cancer *in vivo*. Animal studies demonstrated high efficiency of this novel NPs in PPTT method with the specific parameters of excitation.

## Acknowledgments

This work was financially supported by Shiraz University of Medical Sciences (grant No. 15986). We would like to thank Dr. Ali Mashreghi, a faculty member of Materials Science and Engineering Department, Shiraz University of Technology, Shiraz, Iran, for his helpful comments.

## Ethical Issues

The protocol of the study was approved by Ethical Committee of Shiraz University of Medical Sciences (aproval number 15986).

## Conflict of Interest

The authors declare that they have no conflict of interests. Authors disclose all relationships or interests that could have direct or potential influence or impart bias on the work.
